# Rearrangements in the musculature correlate with jumping behaviour in legless Mediterranean fruit fly larvae *Ceratitis capitata (Tephritidae)*

**DOI:** 10.1038/s41598-022-11369-4

**Published:** 2022-05-06

**Authors:** Max Diesner, Marcel Brenner, Amin Azarsa, Caroline Heymann, Hermann Aberle

**Affiliations:** Institute for Functional Cell Morphology, Heinrich Heine University Düsseldorf, Building 26-12-00, Universitätsstrasse 1, 40225 Düsseldorf, Germany

**Keywords:** Developmental biology, Neuroscience, Zoology

## Abstract

Larvae of holometabolic insects evolved different crawling strategies depending on the presence or absence of larval legs or life style. A rather unusual mode of locomotion has independently evolved in legless larvae of several dipteran species. Maggots of the Mediterranean fruit fly *Ceratitis capitata* developed an effective jumping mechanism to increase locomotion speed or to deter predators during the search for suitable pupation sites. Here, we use high-speed videography to visualize even the fastest movements during jump preparation and take-off. Quantification of kinetic and biometric parameters reveal that maggots jump up to 15-fold of their body length from a standing position and gain speed with 27 times the acceleration of gravity. Videos at high spatial resolution show the mechanism of latch formation and release in unprecedented detail. Mouth hooks insert in the caudal segment and raise a cuticular fold that serves as a handle to pressurize the body prior to launch. Since locomotion behaviour should be intrinsically linked to neuromuscular systems, we dissected third instar larvae and determined the precise pattern of abdominal muscles fibres. Compared to non-jumping dipteran larvae, such as *Drosophila melanogaster*, the overall arrangement is highly similar, but a few muscle fibres show characteristic re-arrangements in orientation and strength that are consistent with a role in bending and jumping. These results suggest that body wall muscles show adaptations to jumping behaviour in *Ceratitis* larvae, and possibly also in other species with different jumping techniques.

## Introduction

Soft-bodied larvae of holometabolic insects employ different modes of crawling in terrestrial environments. Locomotion depends on a hydrostatic skeleton that employs displacements of aqueous fluids in a pressurized body cavity. Supported by unsegmented prolegs on the ventral side of the abdomen, legged larvae (caterpillars) use true legs on thoracic segments for crawling or inching^1,2^. In contrast, legless larvae (maggots) crawl by contraction waves originating in posterior segments^3,4^. Once the peristaltic wave reaches the anterior end, abdominal segments are actually resting on the substrate but the head and interior organs reach forward to translocate the centre of mass^5^. The head and mouth hooks then anchor the larva in the ground, while the next contraction wave begins in caudal segments^5^. Contracting segments shorten and grow thicker, which accentuates their denticle belts, also called creeping welts, that support the crawls by pushing dozens of small, backward-pointing cuticular denticles into the substrate^6^. Because larvae accumulate high amounts of nutrients prior to pupation, and move slow, they are not only prone to desiccation but are also attacked by predators or parasitoids. Larvae of several species have thus evolved specialized behaviours or adaptations to reach their pupation sites safely and rapidly. Responding to a variety of environmental factors, these include, but are not limited to, positive or negative photo-, geo- or hydrotaxis but also characteristic forms of locomotion during the wandering period^7,8^. Some dipteran species including *Piophila casei* (Piophilidae), *Dacus cucurbitae* (Tephritidae), *Mycetophila cingulum* (Mycetophilidae) or *Prochyliza xanthostomata* (Piophilidae) jump out of their hosts to find appropriate pupation sites^9–13^. In comparison to crawling, jumping speeds up locomotion dramatically and is energetically advantageous^14,15^. Ground-based locomotion causes higher costs of transport and is energetically unfavourable in both maggots and caterpillars^16,17^.

The Mediterranean fruit fly, or Medfly, *Ceratitis capitata* (Tephritidae) is a highly destructive, agricultural pest that has adapted to a variety of climatic zones^18^. The polyphagous larvae develop until the third instar in more than 200 tropic and subtropical fruits and vegetables, rendering their pulp unpalatable and producing enormous economic costs^19^. For this reason, the species is also developed as a model organism for pest control by the sterile insect technique^20,21^. At the end of larval development, larvae leave their moist environment to pupate in sandy grounds or cracked soil^22^. During this wandering period, they become vulnerable to desiccation or predation and locomote by particular evading jumps^22^. First, in a process called loop formation, they form an upright loop structure by grapping the cuticle at the posterior end with their mouth hooks^14^. They then compress their body fluids to store elastic energy, before they release the grip, which propels the larva into the air, often covering distances of up to 10 cm and more. Remarkably, young third instar larvae are unable to curl up but learn to jump over time by trial and error^14^. Individual steps in the jump cycle, however, have been difficult to document at the required temporal resolution, due to technical limitations. In addition, the precise mechanisms that trigger latching and launch are still elusive. Recently, larval jumping has been studied in a distantly related species, *Asphondylia sp.* (Cecidomyiidae or gall midges), using a modern high-speed video system with up to 20,000 frames per second^15^. Although these larvae curl into similar ring-like structures (loop formation) and pressurize their bodies before every jump (loop contraction), they utilize a different latching mechanism^15^. Scanning electron micrographs revealed that the latch is formed on a ventral protrusion in the third segment that adheres to the last abdominal segment. The interlocking parts contain bands of cuticular microstructures that seem to form an adhesive strip, similar to Velcro fasteners^15^. Upon latch release, the anterior two-thirds of the larva detaches from the ground, while the posterior part remains on the substrate, forming a "transient leg" that transmits the kinetic energy to the substrate^15^. Based on take-off speeds and horizontal jump distances, energetic comparisons confirmed that jumping is by orders of magnitudes more effective than crawling^15^.

Since the jumping mechanism might differ in various dipteran species, we set out to document it in *Ceratitis capitata* larvae. Using a combination of high-speed imaging and scanning electron microscopy, we drastically improve temporal and spatial resolution, respectively, compared to previous studies^14^, and find that the latch is not formed by cuticular microstructures but by mouth hooks raising a fold in the integument that serves as a handle. Since locomotion in limbless animals is highly dependent on the arrangement of muscle fibres^23^, we dissected larvae and visualized the muscle architecture to correlate it with the jump mechanism. In comparison to non-jumping dipteran larvae, anatomical changes in the orientation and strength of specific muscle fibres seem to facilitate loop formation. The arrangements of muscle fibres might thus be an important prerequisite for jumping, and larvae employing different jump mechanisms might have characteristic reorganizations of contractile fibres.

## Materials and methods

### Ceratitis husbandry and life cycle

**Figure 1:**
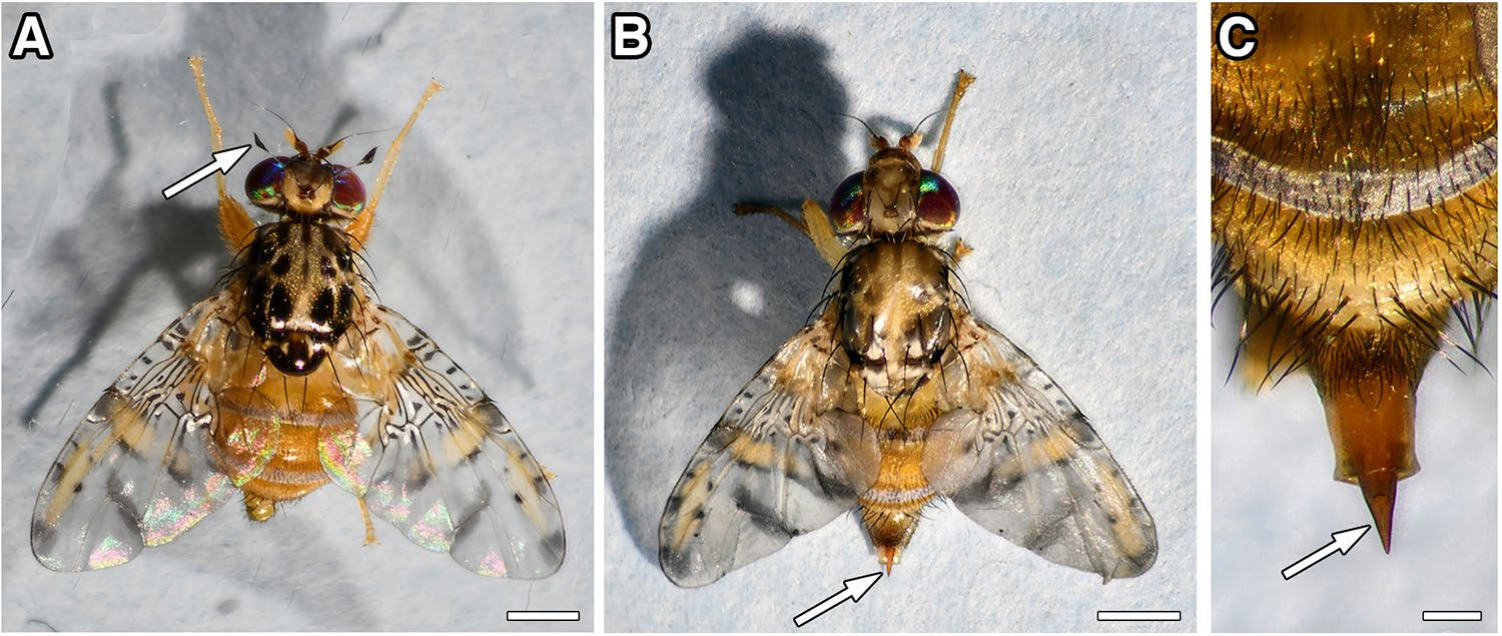
The Mediterranean fruit fly *Ceratitis capitata* is a major agricultural pest and infests a wide variety of fruits and vegetables. The colourful flies are characterized by red eyes, distinct black patches on the scutum and broad, abducted wings with yellowish areas and black spots or stripes. (**A**) Male flies carry sexually dimorphic fronto-orbital bristles with a spatula-shaped terminal end (arrow), which might function as a visual display during courtship (head rocking). (**B**,**C**) Female flies have a sturdy ovipositor at the caudal end (arrow in **B**). Once protracted (arrow in **C**), it is long and sharp enough to deposit eggs in ripening fruits, frequently by penetrating the skin directly or by taking advantage of already existing fractures. Scale bars 1 mm (**A**,**B**), 200 μm (**C**).

*Ceratitis capitata* (Tephritidae) flies have eye-catching spotted wings and peculiar courtship behaviours. Males are identified by dramatically enlarged fronto-orbital bristles (Fig. 1A) that are thought to have a specific display function during courtship in mating arenas, called leks^24^. Females (Fig. 1B) have a long and sturdy ovipositor (Fig. 1C) that is suitable to penetrate the skin of various fruits and vegetables for egg-laying. Developing larvae macerate the pulp until they reach the third instar and leave their hosts. During this wandering phase, larvae search for suitable pupation sites in sandy grounds or dry earth cracks.

Wild-type *Ceratitis capitata* flies (strain EgII) were obtained from Ernst Wimmer (Georg-August-University Göttingen) and Marc Schetelig (Justus-Liebig-University Gießen) and maintained at 23–29 °C (~ 50% rel. humidity, ambient light, 12 h:12 h L/D photoperiod) with access to a saccharose:yeast extract 3:1 and tap water ad libitum. Flies were kept in small cages with one side covered by a gauze. Females used their ovipositor to pierce through the gauze for egg laying. A small petri dish filled with distilled water was placed under the gauze for egg collection. After a 24 h laying period, eggs still attached to the gauze were also collected using a small brush. All eggs were transferred to carrot agar plates^25^ (52.5 g carrot powder, 21.0 g yeast extract, 2.0 g sodium benzoate, 1.75 g agar, 2.25 ml 32% HCl, add 500 ml H_2_0). Nipagin (1.43 g) was added once the boiled food had cooled down to 60 °C. Agar plates were placed in plastic boxes filled with autoclaved play sand as pupation substrate. Boxes were covered with a lid and an additional gauze to prevent crawling and jumping larvae from escaping. Jumping larvae were typically observed after 10–12 days after eggs had been plated. Pupae were collected from the sand using a kitchen sieve and placed in a new cage for hatching. For manual handling, larvae were immobilized on crushed ice by cooling to 2–8 °C, and flies were anesthetized using CO_2_ gas.

### High-speed imaging and photography

Videos were collected at 250 frames per second (fps) or 4000 fps using a 1.3 Megapixel Photron Fastcam (Mini UX100 Model 800 K/M1; VKT Pfullingen, Germany) equipped with an ENEO Megapixel Macro Zoom lens 45–160 mm/F4.5 and a Cosmicar/Pentax X2 Extender (C-Mount, RICOH Imaging Europe). The jumping arena (PVC plastic sheet) was illuminated with a LED lamp (VD7000LP, Vision Devices, Metzingen, Germany). Images were saved on a Lenovo 2447/W530 notebook, processed with Photron Fastcam Viewer software (PFV version 4.0; VKT, Pfullingen, Germany) and converted to MP4 files. Launch from ventral views was recorded by first attaching the Photron camera to a stereo microscope (Stemi 508, Carl Zeiss Microimaging). The setup was then inverted and fixed to a metal stand. A specimen holder with a central opening was placed above the objective of the stereo microscope. A cover slip (60 × 24 mm) in the bottom of plastic petri dish covered by a lid was placed on the specimen holder and illuminated with cold light sources from below (KL-1500 LCD, Schott) and above (LED lamp VD7000LP, Vision Devices).

Positions of mouth hooks during the jump were tracked manually in each selected frame using the Multi Point function in the PFV software package. The resulting graph was copied to Adobe Photoshop (Adobe Systems) and overlaid with representative larval images at corresponding positions. Adult flies were photographed under a stereo microscope (Stemi-508, Carl Zeiss Microimaging) equipped with a single-lens camera (Nikon D850). Single images were taken at various focal planes and combined into a z-stack. Stacks were processed using Picolay software (made available by Heribert Cypionka, http://www.picolay.de) and exported to Adobe Photoshop CS6 (Adobe Systems).

### Quantifications and statistics

Body length was measured along the anterior–posterior axis using Photron Fastcam Viewer software (PFV version 4.0; VKT, Pfullingen, Germany). For weight determination, larvae were transferred into a balanced plastic vial and weighed individually on a precision scale (Kern Abj 220-4NM, Kern & Sohn, Bahlingen, Germany). All jumps were performed on a plastic sheet (ø 5 mm, PVC) and classified in jump preparation (loop formation and loop contraction) and the actual jump phase. Time for loop formation was deduced from high speed movies and lasted from the beginning of lifting to latching (gripping the caudal segment with mouth hooks). Loop contraction was defined from latching to latch release. Take-off time was determined as the interval between latch release and loss of substrate contact. Take-off angle was averaged from the position of the centre of gravity during the first five frames after launch. Since larvae were fully outstretched during flight, the centre of gravity (centroid) was approximated for each animal by measuring larval length and subtracting 50% from the posterior end for any frame evaluated. Take-off speed was calculated from the distances of the centroid during the first five images after launch. Acceleration was defined as take-off speed divided by take-off time. Jump distances were measured between the start position and the landing site using a standard ruler. Videos at 250 fps (n = 40 individual larvae) were evaluated to determine parameters for jump preparation (Table 1). Videos at 4000 fps (n = 8 individual larvae) were used to quantify launch and take-off parameters. All values represent means ± standard deviation (s.d.).

**Table 1:** Kinematic parameters of jumping dipteran larvae.

	*Ceratitis capitata* (Tephritidae)	*Ceratitis capitata* (Tephritidae)	*Asphondylia sp.* (Cecidomyiidae)	*Prochyliza xanthostoma* (Piophilidae)	*Mycetophila cingulum* (Mycetophilidae)
Reference	This study	Maitland et al.^14^	Farley et al.^15^	Bonduriansky^10^	Camazine^11^
Frame rate (fps)	250–4000	25	100–20,000	–	54
Body length (mm)	6.77 ± 0.91	8.50 ± 0.25	3.3 ± 0.3	4–7	8
Body mass (mg)	9.0 ± 3.7	17.0 ± 1.9	1.3 ± 0.3	–	–
Loop formation (s)	1.43 ± 0.22	0.9 ± 0.1	1.8 ± 0.3	–	–
Loop contraction (s)	1.04 ± 0.23	0.7 ± 0.1	1.6 ± 0.8	–	0.4 ± 0.1
Jump preparation (s)	2.47 ± 0.34	~ 1.6 ± 0.2	3.4 ± 1.1	–	–
Take-off time (ms; n = 8)	3.29 ± 0.48	1.37	1.2 ± 0.2	–	–
Take off angle (°; n = 8)	71 ± 5	60	63 ± 3	–	69.3 ± 8.5
Take-off speed (m/s; n = 8)	0.87 ± 0.20	1.17	0.85 ± 0.20	–	~ 0.85–1.58
Acceleration (m/s^2^; n = 8)	~ 264 (~ 27 g)	~ 854 (~ 86 g)	880 ± 220 (~ 90 ± 22 g)	–	~ 234 (~ 24 g)
Jump distance (mm)	100 ± 38	120 ± 8	77 ± 12	200–500	95 ± 28
Jump distance (fold body length)	~ 15-fold	~ 14-fold	23.1 ± 3.6-fold	–	24-fold
Latch	Mouth hooks	Mouth hooks	Cuticular microstructures	Mouth hooks	Cuticular microstructures
Latch formation	Ventral side	Ventral side	Ventral side	Ventral side	Dorsal side

Data are means ± s.d., if available. Dash, data not reported. Our study n = 40, except where indicated.

### Larval dissection and immunohistochemistry

Dissection of *Ceratitis* and *Drosophila* larvae was adopted from^26^. *C. capitata* larvae were collected at the third instar stage and tested for jumping capability. Larvae were washed in ice-cold PBS, pinned on Sylgaard plates (Dow Chemicals) using minutien pins (Emil Arlt) and dissected in ice cold PBS. Larval fillets were rinsed several times with fresh PBS to remove remaining tissues and fixed with 3.7% paraformaldehyde (PFA) for 2 × 5 min and subsequently for 15 min at RT. Fixative was removed by rinsing 3× with fresh PBS and fillets were transferred into a plastic vial containing PTx (PBS, 0.1% Triton-X100). Fillets were permeabilized 4 × 15 min on an orbital shaker using PTx. If better tissue penetration was desired, the first permeabilization wash was performed in PBS/1% SDS for 5 min. Unspecific binding sites were blocked by incubation in PTx/5% NGS (normal goat serum) for 90 min at RT on an orbital shaker. Phalloidin coupled to TexasRed (1:2000, Molecular Probes, now Thermo Fisher Scientific) was added for 90 min at RT. Fillets were washed four times with PTx and cleared in PBS/70% Glycerol overnight at 4 °C. Head and tail regions were cut under a dissecting scope using scissors (Fine Science Tools, No. 15018–10), and fillets were mounted in PBS/70% Glycerol on microscope slides for imaging.

Microscopic images were acquired using a laser-scanning confocal microscope (LSM710, Carl Zeiss MicroImaging) equipped with air objectives (20×/0.8 and 40x/0.95Korr Plan Apochromat). Images (1024 × 1024 pixel, line averaging 2) were processed using Fiji is just ImageJ, version 1.52 s^27^ and Adobe Photoshop CS6 (Adobe Systems). Figures show maximum intensity projections of several z-sections. Tile scans are indicated in figure legends.

Schemes of the musculature were created using Graphpad Prism (Graphpad Software) connected to Adobe Illustrator (Adobe Systems) and a printed sample image as template.

### Scanning electron microscopy (SEM)

Larvae were snap frozen prior to launch using liquid nitrogen, fixed in Karnowsky fixative^28^ and dehydrated in an ascending ethanol series (50%, 70%, 80%, 90%, 96%). Ethanol was replaced by washing larvae sequentially in ethanol:acetone 3:1, 1:1, 1:3 and acetone only. Larvae were transferred to Acroseal (99% Tetramethylsilan; Acros Organics, Germany) by washing them sequentially in aceton:Acroseal 1:3, 1:1, 3:1. Larvae were completely desiccated by evaporation under a fume hood, sputtered with a gold layer and prepared for SEM.

## Results

### Jump preparation in *Ceratitis capitata* larvae

*Ceratitis* larvae acquired the ability to jump at the end of the third instar stage. Jumping was not performed by immature stages but was obviously exercised by fed larvae that had to practice loop formation several times before being able to jump. Similar observations have been reported for piophilid carrion flies^10^. Jumping in *Ceratitis* was subdivided into a preparatory phase and jumping phase^14^. The preparatory phase itself consist of two successive steps. First, loop formation, where the larva erects itself into a ring-like structure. And second, tension generation or loop contraction, where muscle contraction sets this ring under tension^14,15^.

**Figure 2:**
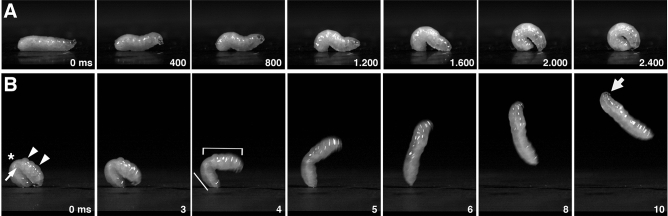
Jump preparation comprises two major steps in legless *C. capitata* larvae: Loop formation and loop contraction. (**A**) Loop formation consists of two phases: lifting and latching. With the head raised, larvae lift themselves into an upright, loop-like figure that is stabilized by planting both the head and caudal segment solidly on the substrate (800–1600 ms). During latching, mouth hooks insert firmly into the protruding ventral portion of the caudal segment, which closes the loop (2000–2400 ms). (**B**) During loop contraction or tension generation, larvae flatten (asterisk) and kink (arrow) at the most exposed region, which causes bulging and thickening of the anterior end (arrowheads, 0 ms). Take-off is triggered by mouth hook release (3 ms). Anterior segments (bracket) snap into the air, while the posterior end (white line) transduces the kinetic energy to the substrate (4–5 ms). Larvae fly forward but spin backward. Head and mouth hooks are retracted during flight (thick arrow).

To resolve specific features during jump preparation and jumping at high spatial and temporal resolution (1024 × 1024 pixel, 250–4000 frames s^−1^), we placed third instar larvae on a flat PVC plastic sheet as a substrate and imaged their behaviour using high speed videography (Fig. 2, Suppl. Movie S1). We divided loop formation into two distinct phases: lifting and latching. During lifting, larvae raised their head and erected themselves by muscular contractions in the posterior third of the body, which resulted in a wave-like posture (Fig. 2A, 800–1200 ms). Larvae then withdrew their mouth hooks and used the blunted head region to push the provisional bulge higher (Fig. 2A, 1600 ms). With the head and tail regions now firmly placed on the substrate, larvae were ready for latching. To close the loop, the head moved parallel to the substrate towards the protruding ventral portion of the posterior end (Fig. 2A, 2000 ms). The loop was stabilized by the mouth hooks grabbing the posterior end, which was inclined by an angle of about 47° and stably rested with its dorsal cuticle on the substrate (Fig. 2A, 2400 ms). Ventral body surfaces, in contrast, almost entirely lost contact to the substrate and were tightly apposed along a cleft between the anterior and posterior parts of the body (Fig. 2A, 2400 ms). Loop formation was the slowest step of the preparatory phase and took on average 1.43 ± 0.22 s until complete (n = 40; Table 1).

Next, tension was generated by muscle contractions that flattened dorsal surfaces near A4 and A5 and displaced hemolymph both anteriorly and posteriorly, leading to a thickening and bulging of the head and tail regions (Fig. 2B, 0 ms; Suppl. Movie S2). Consequently, ventral surfaces converged even more tightly, creating a kink on the ventral side between A5-A6. The mouth hooks continued pulling on the latch at the caudal segment, which still withstood the traction. Loop contraction lasted for about 1,04 ± 0.23 s, elevating the total time for jump preparation to 2.47 ± 0.34 s (n = 40; Table 1). The latch was finally released by disengaging the mouth hooks (Fig. 2B, 3 ms). During the first milliseconds of take-off, the two-thirds of the larvae soared into the air, while, in a brief but powerful stroke, the protruding caudal segment snapped heavily onto the substrate to move the centre of gravity forward and to trigger the jump in a forward direction (Suppl. Movie S2, 2.00–2.75 ms). Positioning of the posterior end has thus a major impact on jump performance and take-off.

**Figure 3:**
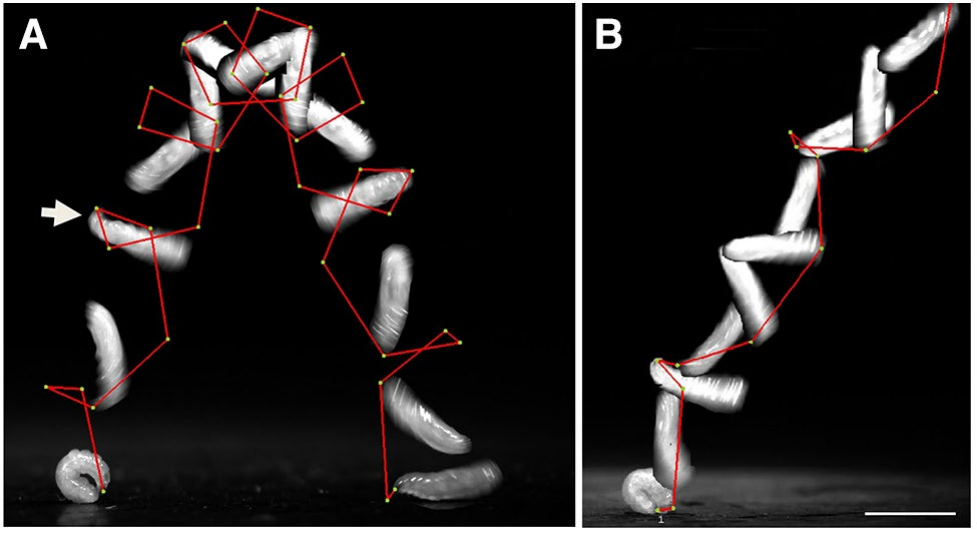
Jumping *Ceratitis* larvae rotate in backward somersaults along a ballistic trajectory. (**A**) Stop motion images taken from a steep but short ballistic jump overlaid with the positions of the mouth hooks (green dots) in selected frames. Positions between frames were deduced (red line). Head and mouth hooks are bent forward (thick arrow). (**B**) Less steep take-off angles result in longer jump distances. Scale bar 5 mm.

### Launch and kinematics of the jump phase

While the posterior end of the larva was still in contact with the substrate, the whirling anterior part fuelled the jump (Fig. 2B, 4–5 ms). During this thrust phase, the anterior head region could be compared to a lever arm that rotates around the kink (pivot point) to transmit its force and kinetic energy to the load arm (posterior end) and thus the substrate. As soon as the body was completely straight, the larva lost contact to the substrate (Fig. 2B, 6 ms). Take-off time, the time between latch release and lift-off, was extremely fast and took only 3.29 ± 0.48 ms (n = 8). The larva left the ground at an average angle of 71 ± 5° (take-off angle), rotating backwards around its centre of gravity (Fig. 2B, 8–10 ms). Thus, the inclined posterior end and the soaring anterior end together cause the larva flying forward in backward somersaults.

**Figure 4:**
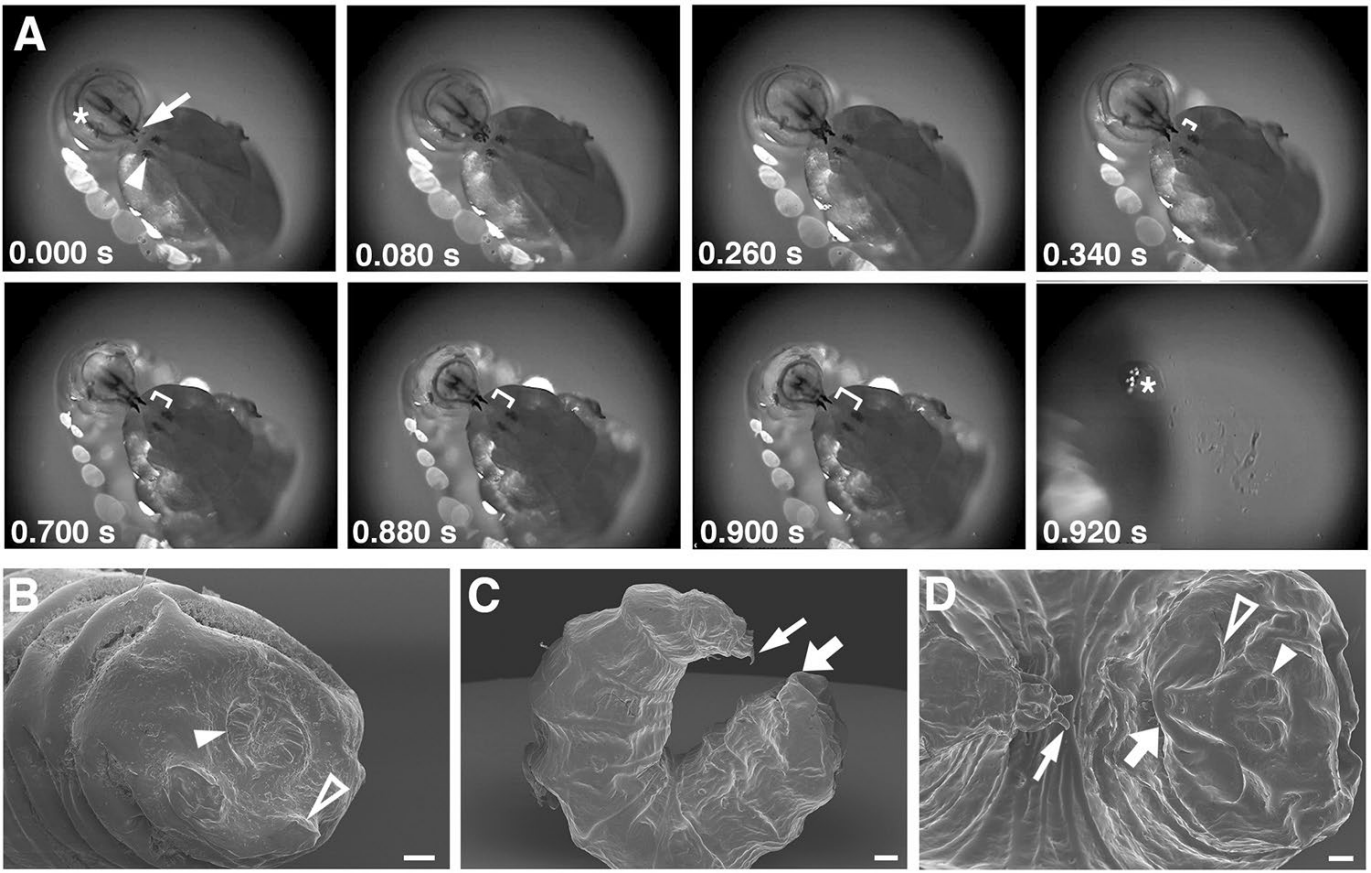
Mouth hooks insert into an epidermal fold during latching. (**A**) Ventral views of a launching *C. capitata* larvae under a stereo microscope equipped with a high speed video camera. Mouth hooks (arrow) insert in the caudal segment just below the posterior spiracles (arrowhead). During tension generation (0.08–0.90 s), mouth hooks slide downward, increasing the distance to the spiracles (bracket) and creating a rimmed fold in an epidermal lamella (Suppl. Movie S3). Mouth hook release and take-off last less than 20 ms (0.92 s). Asterisks mark residual liquid used for cleaning the larva. (**B**–**D**) Scanning electron microscope (SEM) images of *C. capitata* larvae frozen in liquid nitrogen during crawling (**B**) and shortly before launch (**C**,**D**). Arrowheads mark the posterior spiracles, open arrowheads mark the caudal ridge. Lateral (**C**) and ventral (**D**) views of the same larva just after freezing-induced mouth hook release. Arrows label the extended mouth hooks, thick arrows mark the epidermal fold (latch). Mouth hooks pull on this fold and their imprints are still visible. Scale bars 100 μm (**B**,**D**), 200 μm (**C**).

**Figure 5:**
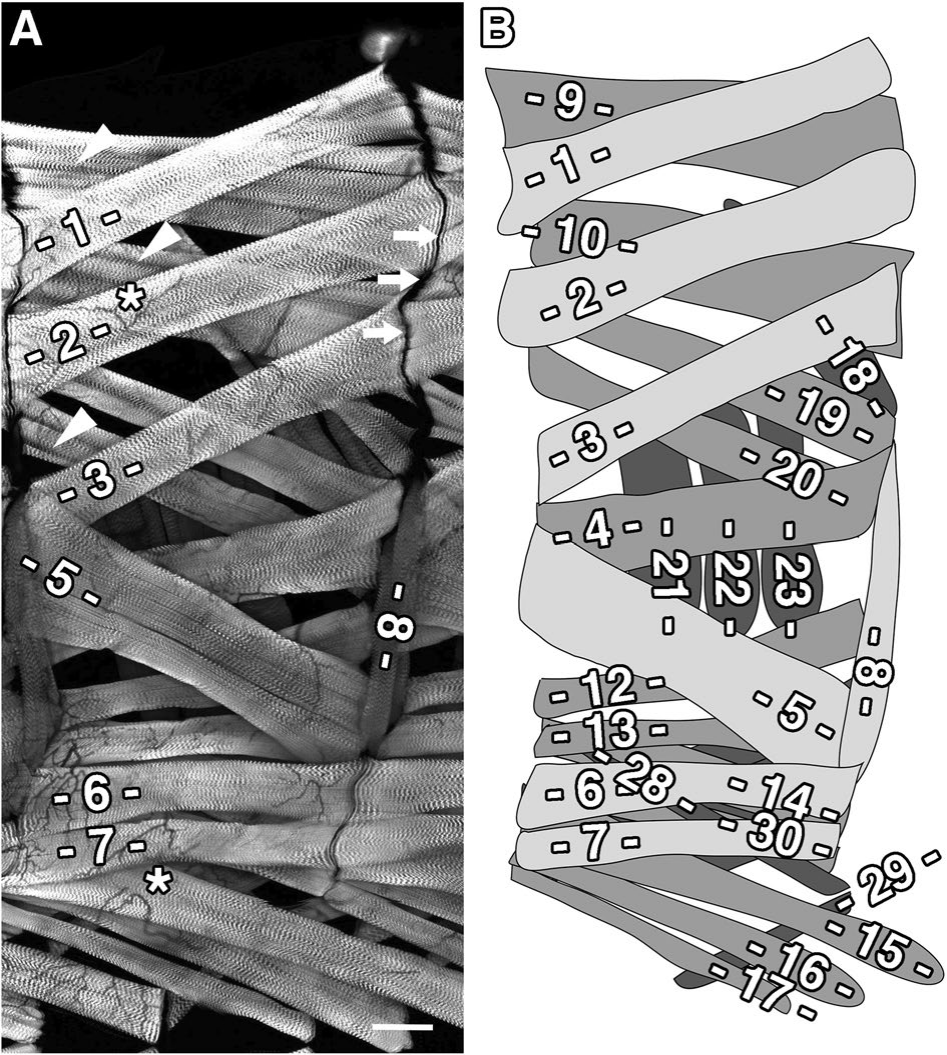
Internal muscles in abdominal segment A3 of a *C. capitata* larva. (**A**) Confocal image (projection) of abdominal segment A3 in a dissected third instar larva prior to the jumping phase and stained with phalloidin to label actin filaments in sarcomeres. Internal muscles M1–M8 are indicated. M5 is particularly massive and clearly conceals M4, which has thus been assigned to the medium layer. Arrows highlight posterior muscle attachment sites, arrowheads show "splitting" of muscle fibres, asterisks indicate areas with tracheal branches. (**B**) Corresponding scheme of the musculature. Internal muscles (light grey), medial muscles (dark grey) and external muscles (black). Muscle fibres are numbered based on their position and orientation. Not all muscle fibres labelled here are visible in the image shown in (**A**). Dorsal is up and anterior left. Scale bar 100 μm.

**Figure 6:**
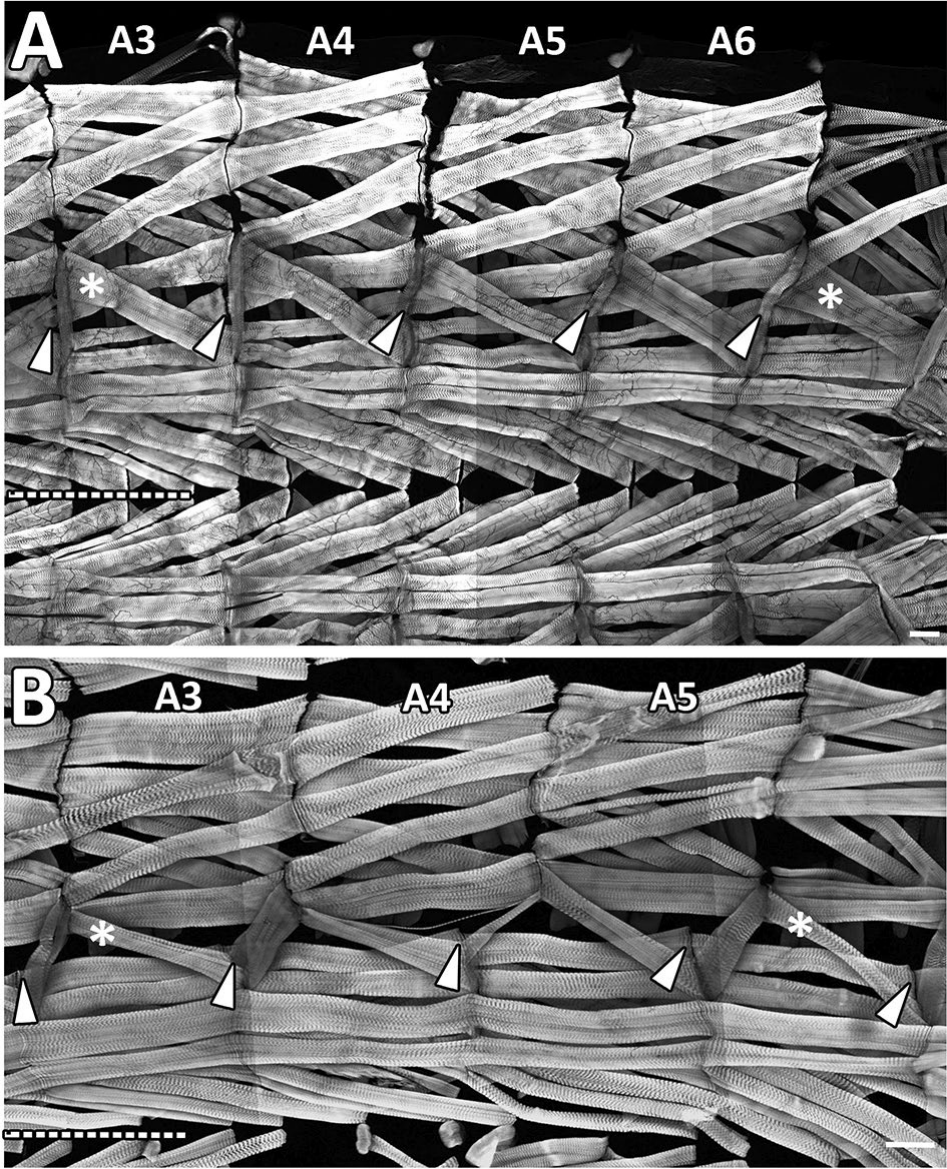
Comparison of the stereotyped muscle pattern in abdominal segments of *Ceratitis* and *Drosophila* larvae. (**A**) Tile scan of a dissected *C. capitata* larva showing phalloidin stained muscles fibres in abdominal segments A3–A6. M5 (asterisks) is particularly thick in all segments. Arrowheads indicate that M12 joins the attachment site of M4 in the next posterior segment. Dashed line labels the ventral midline. (**B**) Tile scan of a dissected *Drosophila* larva showing muscles in abdominal segments A3–A6. Arrowheads indicate that M12 joins the attachment site of M12 in the next posterior segment. M5 (asterisks) is relatively thin, but ventral longitudinal muscles (M12, M13, M6, M7) are comparatively strong, which is probably advantageous for crawling on horizontal substrates. Dorsal is up and anterior left. Note different scales. Scale bars 100 μm.

Without having any legs, *Ceratitis* larvae jumped on average 100 ± 38 mm (n = 40) from a standing position under our conditions (Table 1). Considering a body length of 6.77 ± 0.91 mm, this equalled approximately 15 times their body length (Table 1). Converted to humans with a supposed average height of 1.75 m, this would equal approximately 26 m, far more than a long jumper (with legs) could achieve. We also determined the speed at launch by relating the advance of the calculated centre of gravity in the first five video frames, resulting in an average take-off speed of 0.87 ± 0.20 m/s (n = 40). It is interesting to note that larvae were able to jump several times in a row (up to 11 times per min), confirming previous observations^14^. Considering a maximal crawling speed of 0.2 cm/s (= 12 cm/min)^14^, several jumps in a row is evidently faster than crawling, even if a straight direction is usually difficult to achieve in consecutive jumps. In fact, when energetic and metabolic costs were considered, legless jumping was clearly more efficient on solid substrates than legless crawling^15,17^.

A previous study has shown that *Asphondylia* larvae are slightly curved during flight^15^. To analyse body postures in *Ceratitis* larvae, we created still frames of jumping larvae and tracked the positions of their mouth hooks in regular intervals (Fig. 3). Take-off angle and flight trajectory were generally variable, but a steeper take-off angle resulted in a steeper trajectory and a shorter jump distance (Fig. 3A). The variability in jump height and distance depended mostly on the positioning of the larvae on the substrate during loop formation (Suppl. Fig. S1). However, inclination angle, narrowness of the cleft or mouth hook release also seemed to play a role. Body postures were very stable during flight, and larvae were fully stretched at all time points. Speed became slower towards the vertex and accelerated again during descent (Fig. 3A). Positioning of the head during flight was interesting, as it was not sticking out, but was safely ducked on the ventral cuticle (thick arrows in Figs. 2B and 3A). Less steep flight trajectories resulted in longer jumps, but we failed to record them in their entire length as larvae flew out of the focal planes (Fig. 3B).

### Latching in *Ceratitis capitata* larvae

The latching mechanism is probably also an important determinant of jump kinematics and efficiency. Previous studies proposed adhesive microhairs or finger-like microstructures that function as latches in *Asphondylia* or *Mycetophila* larvae^11,15^. Tephrid or piophilid larvae, in contrast, anchor their mouth hooks in the posterior integument just below the posterior spiracles^10,14^. However, microscopic images of locked latches are still lacking and no cuticular specializations or adhesive structures have been found in scanning electron micrographs (SEM) of *C. capitata* larvae or other species in this genus^29^.

We therefore imaged latching at high temporal and spatial resolution by installing a high-speed video camera on a dissecting scope to capture the precise positions of the mouth hooks during launch (Fig. 4, Suppl. Movie S3). Upon approaching the caudal segment, larvae extended their mouth hooks out of the oral cavity (Fig. 4A). The mandibles first grabbed the integument just beneath the posterior spiracles but above the caudal ridges (for anatomical terms see Fig. 4B–D)^30,31^. Mouth hooks then glided slowly downward, continuously increasing the distance to the spiracles (brackets in Fig. 4A). Due to an epidermal lamella between the left and right caudal ridge, downward sliding of the mouth hooks induced a wrinkle in the epidermis that served as a handle for the mouth hooks (Suppl. Movie S3). After a brief period of apparent cessation, mouth hooks suddenly slipped over the wrinkle and the larvae launched into the air. Launch was thus triggered by the inability of the bump to withstand the pulling forces of the mouth hooks. We were unable to see any movements of the mouth hooks, contractions of muscle groups or an anteriorly-directed wave of muscle contractions^14^ prior to launch. Rather sliding over the bulge appeared to trigger strain release, suggesting that cuticular friction plays a central role.

To better visualize cuticular structures in the caudal segment, we submerged *Ceratitis* third instar larvae in liquid nitrogen and prepared them for scanning electron microscopy (SEM). In crawling larvae, posterior spiracles and a thickened cuticle at the caudal ridge were the most noticeable structures of the caudal segment (Fig. 4B). When we submerged looped larvae in liquid nitrogen, just prior to launch, the latch opened unfortunately, but the overall posture was well preserved (Fig. 4C). The gripping mouth hooks were still extended, and the wrinkle in the epidermal lamella was still discernible and displayed striking imprints of the mouth hooks (Fig. 4D). Thus, the latch is formed by a stretchable epidermal barrage rather than adhesive cuticular microstructures.

### The larval muscle pattern of *Ceratitis capitata*

Since loop formation, tension generation and jumping are all directly or indirectly powered by muscle contractions or relaxations, we were wondering whether *Ceratitis* larvae exhibited a particular muscle pattern that distinguishes them from non-jumping larvae, such as *Drosophila*. Based on earlier descriptions, Crossley noted that cyclorraphan larvae, a group of flies that include the genera *Ceratitis*, *Piophila* and *Drosophila*, possess a rather similar muscle architecture^32^. Some of these larvae, however, exhibit quite different locomotion patterns—crawling and jumping. Are there alterations in the muscle pattern that correlate with these different types of movement?

Since we were unable to find any description of the organization of body wall muscles for jumping larvae, we dissected *Ceratitis* third instar larvae and stained the resulting fillet preparations with fluorophore-conjugated phalloidin, which highlights filamentous actin in muscle fibres and other cell types across the animal kingdom. Number and orientation of muscle fibres were highly similar in abdominal hemisegments and arranged in mediolateral layers (Fig. 5). Phalloidin prominently revealed the sarcomeric pattern but did not stain air-filled tracheal branches stretching along the muscle surfaces (asterisks in Fig. 5A). To describe individual muscles, we adopted our terminology to the nomenclature developed for *Calliphora* and *Drosophila*^32–34^.

The inner-most layer consisted of oblique muscles in dorsal (M1, M2, M3) and lateral (M5, M8) regions and longitudinal muscles in ventral areas (M6, M7). Both types of fibres were inserted in specific attachment sites at segment borders (arrows in Fig. 5A). Muscle 8, frequently called segment border muscle, is perfectly oriented along these sites (Fig. 5A). In the intermediate layer, muscles M9 and M10 were the dorsal-most fibres, while M4, M19 and M20 represented the dorso-lateral area and M12-M17 and M30 the ventral region (dark grey in Fig. 5B). We grouped muscle 4 into this layer, because it was prominently occluded by muscle 5. A variable subset of muscles in all layers contained deep longitudinal grooves, which divided a single fibre into several smaller fibres, at least partially (arrowheads in Fig. 5A). This "splitting" was most prominent near attachment sites but spread occasionally along the entire length of a muscle fibre.

The external layer, closest to the cuticle and shaded black in Fig. 5B, lacked longitudinal muscles altogether. Muscle 18, a transverse muscle in the dorsal region, was highly variable and appeared as a bundle of three densely packed, individual muscle fibres. In the lateral muscle field, M21-M23 formed three prominent transverse fibres. It is interesting to note that we failed to identify a fourth transverse fibre, M24, which is known in *Drosophila*. Muscle 11 was similarly absent in abdominal segments A2-A7 (see below and Suppl. Fig. S2). It is formerly possible that M11 and M24 became part of multi-fingered M18 during embryonic development but this awaits further testing. Superficial muscles in the ventral region consisted of oblique fibres M25-M29. As in *Drosophila*, M29 stretches from transboundary muscle M15 to its attachment site at the posterior segment border.

While abdominal segments A3–A6 were highly stereotypic, A1–A2 and A7 diverged slightly from this pattern. First, A1 contained an additional oblique muscle M11 (Suppl. Fig. S2), and M18 was composed of only two fibres. Second, A2 contained a triangular fibre, external to and tightly associated with muscle 6 that projected to M8 and caused M6 to appear much wider (Suppl. Fig. S3). Third, in A7, M1, M2 and M9 converged at almost a single attachment site in the dorsal posterior segment border (Suppl. Fig. S4).

Thus, while the *Ceratitis* larval muscle pattern was highly similar to *Drosophila* larvae in terms of fibre number and orientation, we noted some important differences that might be relevant for jump preparation, when we compared several segments in a row (Fig. 6). Most importantly, oblique M12 was not apposed to M12 in the next, posterior segment, as seen in *Drosophila*, but shared its attachment site with the more dorsally positioned M4 (compare arrowheads in Fig. 6A,B). In general, longitudinal muscles were thinner in *Ceratitis*, while oblique muscles dominated. This was best visible at M5 (asterisks in Fig. 6) but was also obvious for M3. A distinctive feature was also M13 that, in contrast to *Drosophila*, became thicker towards the posterior end, while M6 and M7 got progressively thinner (Fig. 6). Lastly, M20, which in *Drosophila* is somewhat rudimentary and bent, was well developed in *Ceratitis*. This comparison indicates that a slight reorganization of the muscle pattern, in particular a steeper projection of M12 and the dominance of oblique muscles, might facilitate loop formation and jumping in *Ceratitis* larvae.

## Discussion

We describe the precise jumping process in legless *Ceratitis capitata* larvae and document an alternative latching mechanism. Larvae at the jumping stage erect themselves into a loop by "standing" on head and tail segments. When the mouth hooks latch on the caudal segment and pull against it, the loop is pressurized by muscle contractions. Upon latch release, stored energy is transmitted to the substrate by a catapulting mechanism, which launches the larvae into the air. Take-off angles and flight trajectories were quite variable, as positioning of the posterior end on the substrate and achieving the optimal buckled posture during tension generation impacted jump performance.

While many kinematic and biometric studies have been performed with leaping and creeping insect larvae, high resolution movies of crawling or jumping larvae are still scarce. Because jumping is extremely fast, previous studies were largely limited by temporal resolution. Reduced imaging speed could hence be a reason for differences in kinetic parameters, which we have observed in comparison to an earlier study, such as take-off time or take-off speed^14^. Another reason for significant differences in body weight and length could be different food sources for raising *Ceratitis* larvae^14,25^.

Similar to our study, only one other study, to our knowledge, used modern high-speed videography to resolve even the fastest movements during launch in jumping dipteran larvae^15^, giving us the opportunity to compare two different species. While there are many similarities in jumping *Ceratitis* and *Asphondylia* larvae, there are also a few notable differences. First, although larvae in both species follow ballistic trajectories, *Asphondylia* larvae maintain a slight curvature during flight, whereas *Ceratitis* larvae showed a straight posture, with the transient hinge completely straightened^15^. Second, the latch is located at different body positions. In *Asphondylia*, it is located on a ventral protrusion in the third segment, which results in an imperfect loop structure^15^. Third, latching employs entirely different mechanisms. In *Asphondylia*, it does not require mouth hooks or the spatula, a special cuticular structure used to exit the gall, but relies on adhesion of opposed body parts. The protrusion on the ventral surface carries cuticular microstructures that attach to similar structures in the caudal segment during loop formation^15^. In contrast, *Ceratitis* employs its mouth hooks for latching. The mouth hooks grasp a cuticular lamella below the posterior spiracles in the terminal segment. The resulting loop covers therefore the entire length of the larva. Interestingly, when the mouth hooks pull on the lamella, it converts into a formidable grip. The lamella is not visible prior to loop formation but arises when the mouth hooks slide downward during tension generation. At maximal tension, it is unable to withstand the pulling forces and releases the strain, when mouth hooks slide over it. The trigger mechanism might in fact be analogous to snapping of a finger in humans, where the fingertips form a compressible latch that is released by overcoming skin friction^35^. Compared to adhesive latching in *Asphondylia*, *Ceratitis* larvae might thus employ frictional latches.

Jumping cheese borer larvae, *Piophila casei*, have also been reported to use a cuticular flap at the caudal segment (located between two ventral conical pegs) for hooking their mandibles^9^. A related piophilid species, *Prochyliza xanthostoma*, similarly inserts its mouth hooks in a region of the caudal segment during jump preparation^10^. It is therefore possible that all three species use similar latching mechanisms but high-resolution images of the posterior end at launch are still lacking.

Precise muscle contractions control much of jumping and jump preparation. While various descriptions of jumping larvae exist^29,36–38^, the underlying muscular system was not examined and has been reported only for non-jumping dipteran larvae^33,39,40^. We assumed that the type of movement and the underlying muscle pattern could be intrinsically linked, and differences in locomotion between jumping and non-jumping larvae might be reflected in the anatomy. To search for alterations in number, strength and orientation of muscle fibres, we dissected third instar *Ceratitis* and *Drosophila* larvae. The muscle patterns were highly similar but showed two notable exceptions. First, muscle 12 was connected with muscle 4 at posterior segment borders, thereby linking the ventral with the lateral muscle field. Contractions of this muscles might thus facilitate downward bending over segment shortening. Second, compared to longitudinal muscles, oblique fibres seemed to dominate in *Ceratitis* larvae, with muscles 3 and 5 being clearly thicker than in non-jumping *Drosophila* or *Calliphora*^33^ larvae. Contraction of muscle 5 probably supports downward bending, too, since ventral surfaces shrink during loop formation, while dorsal surfaces increase. This suggests that dorsal-most muscles and muscle 3 are likely stretched during loop formation but snap back during take-off to support the catapulting upward motion. Oblique muscles might thus contribute to elastic energy storage and power the jump.

Longitudinal muscles, in contrast, appeared underdeveloped in *Ceratitis* larvae, when compared to *Drosophila* larvae, especially on the ventral side. Longitudinal muscles seem to dominate in non-jumping *Calliphora* larvae as well^33,40^. In particular, muscles 6 and 7 grow to astonishing thicknesses^40^. One reason for these differences might be that *Ceratitis* larvae mostly reside within their hosts and do not crawl over extended distances. Since longitudinal muscles are mainly required for horizontal crawling, they might be less used in *Ceratitis* and remain smaller.

In jumping larvae, muscles contractions hold the body under intense tension before launch. While muscle fibres with their contractile filaments might contribute to the sudden energy release, elastic energy is certainly stored in the expandable fibre networks of connective tissues and/or the non-sclerotised cuticle, as displacements of incompressible body fluids during tension generation dilate body parts and extracellular matrices^41^. The anterior and posterior end of *Ceratitis* larvae clearly swelled during jump preparation due to contraction-induced displacement of hemolymph along the body cavity, which was completely restored during and after the jump.

It has been noted previously that bulges and kinks are usually not observed in cylindrical animals with pressurized hydrostatic skeletons^42^. Since jumping larvae undergo obvious kinking and since the "kink" remains visible, at least in *Asphondylia*, during flight^15^, we wondered if a particular muscle architecture facilitates kinking. However, since this constriction occurs between segments A4 to A6, which are highly stereotypic, we could not detect specific differences in the muscle pattern that would readily explain the formation of this constriction. In this respect, it is interesting that *Asphondylia* and *Mycetophila* larvae use an entirely different looping mechanism. While *Asphondylia* loops along its ventral side, *Mycetophila* curls back to attach on its dorsal side^11^. It would thus be interesting to see if these opposed looping mechanisms are reflected in different muscle patterns.

## Supplementary Information

Supplementary Information 1.

Supplementary Video 1.

Supplementary Video 2.

Supplementary Video 3.
