# Supplementary material for: Rearrangements in the musculature correlate with jumping behaviour in legless Mediterranean fruit fly larvae Ceratitis capitata (Tephritidae)
Source: Sci Rep. 2022 May 6;12:7457. doi: 10.1038/s41598-022-11369-4 (PMC9076836; doi:10.1038/s41598-022-11369-4)
Supplement: Supplementary file 1 — Supplementary Information 1. [file 41598_2022_11369_MOESM1_ESM.pdf]

Supplementary Information to:

Rearrangements in the musculature correlate with jumping behaviour in legless Mediterranean fruit fly larvae *Ceratitis capitata* (Tephritidae)

by

Max Diesner[1], Marcel Brenner[1], Amin Azarsa[1], Caroline Heymann[1] and Hermann Aberle[1,*]

[1]Heinrich Heine University Düsseldorf, Institute for Functional Cell Morphology, Building 26-12-00, Universitätsstrasse 1, 40225 Düsseldorf, Germany

Descriptions of Supplementary Movies

Suppl. Movie S1 Loop formation in *C. capitata*.

Loop formation commences with the larvae raising its head and adopting a wave like figure. Next, the larva lifts itself by positioning the head and caudal segments on the substrate, which generates a U-shaped figure. Note that the mouth hooks are retracted during lifting. The loop is closed by the head moving parallel to the substrate and inserting the mouth hooks in the protruding edge of the caudal segment. Frame rate: 250 fps, 4 ms per frame.

Suppl. Movie S2 Launch of a *C. capitata* larva (lateral view).

Prior to launch, the larva is clearly kinked with a flattened body near the cleft and bulged anterior and posterior ends. During launch, the caudal segment snaps onto the substrate (2.00-2.75 ms), while the anterior region swings into the air. This moves the centre of gravity forward. Head and mouth hooks are not extended but huddled against the ventral cuticle. Once airborne, the larva maintains a straight posture and advances anteriorly in backward somersaults. Frame rate: 4000 fps, 0.25 ms per frame.

Suppl. Movie S3 Latching and launch of a *C. capitata* larva (ventral view).

During latching, mouth hooks grab the caudal segment beneath the posterior spiracles. They then slide ventrally, increasing the distance to the spiracles, which creates a ridge in the integument. The mouth hooks slip over the rim and the larva launches into the air. Note reduced playback speed at the end of the movie and receding liquid around the head. Frame rate: 2000 fps, 0.5 ms per frame.

Supplementary Figures

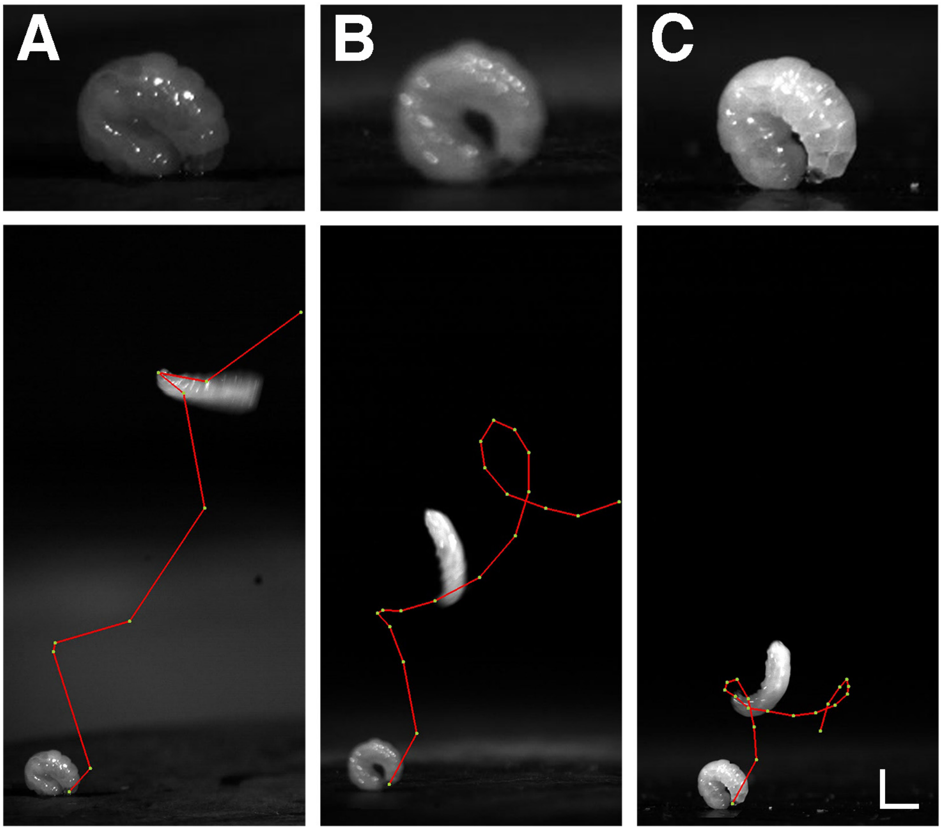

Suppl. Fig. S1

Supplementary Fig. S1

Stop motion images of *C. capitata* larvae showing variability in jump performance depending on positioning of the caudal segment and loop closure. (A-C) Starting position (upper row) and flight trajectory (lower row) of three different larvae. Green dots mark the position of mouth hooks at every frame (4 ms/frame, 250 fps). Red line arbitrarily connects green dots to approximate the flight trajectory.

A) For an average jump, the inclination angle (a virtual line between the ventral body surfaces and the substrate) was 47° ± 5° (n = 46) and the loop was completely closed, i.e. the ventral body surfaces were tightly apposed. B) Wide and open loop structures resulted in lower and shorter jumps. C) Failed jumps correlated with steeper inclination angles (>55°) or premature slippage of the mouth hooks.

Scale bar 2,5 mm.

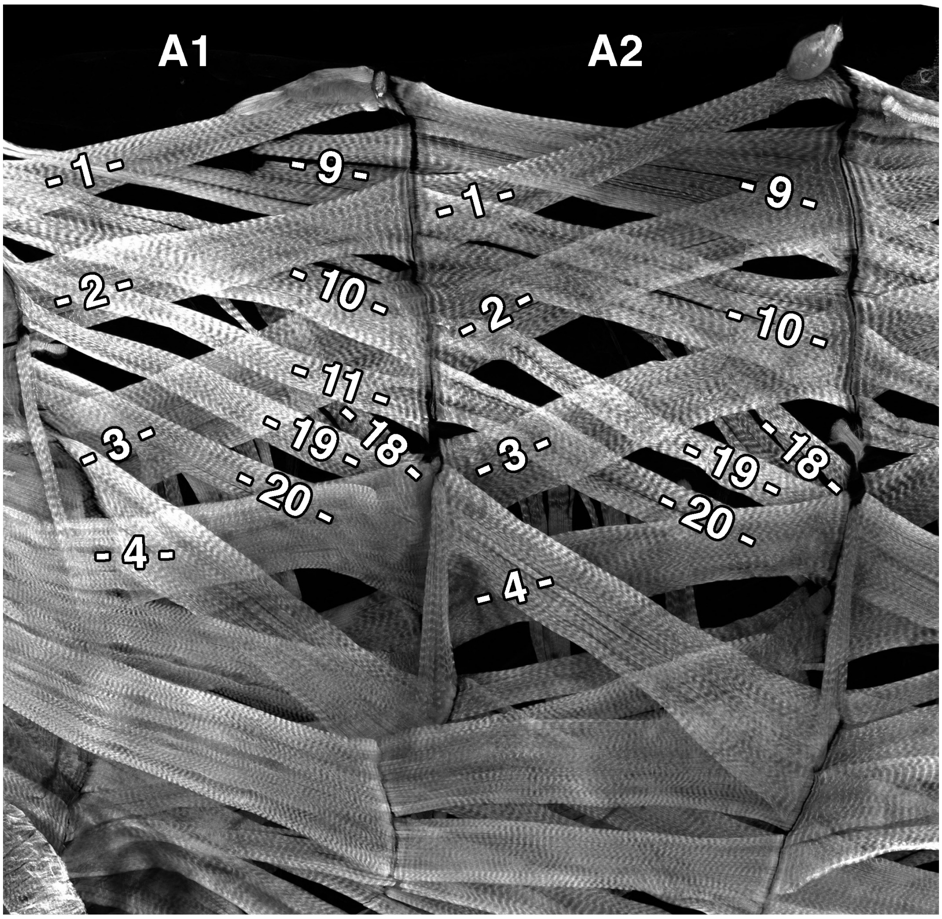

Suppl. Fig. S2

Supplementary Fig. S2

Dorsal muscle pattern in abdominal hemisegments A1 and A2 of a *Ceratitis capitata* larva.

(Left) Confocal image (projection) of A1 in a dissected mid-stage third instar larva stained with phalloidin to label sarcomeric actin filaments.Among medial oblique muscles, M11 is clearly visible as a single fibre.

(Right) Muscle pattern in abdominal hemisegment A2, which lacks M11.

Dorsal muscle fibres are numbered based on their position and orientation according to the *Drosophila* nomenclature. Dorsal is up and anterior left.

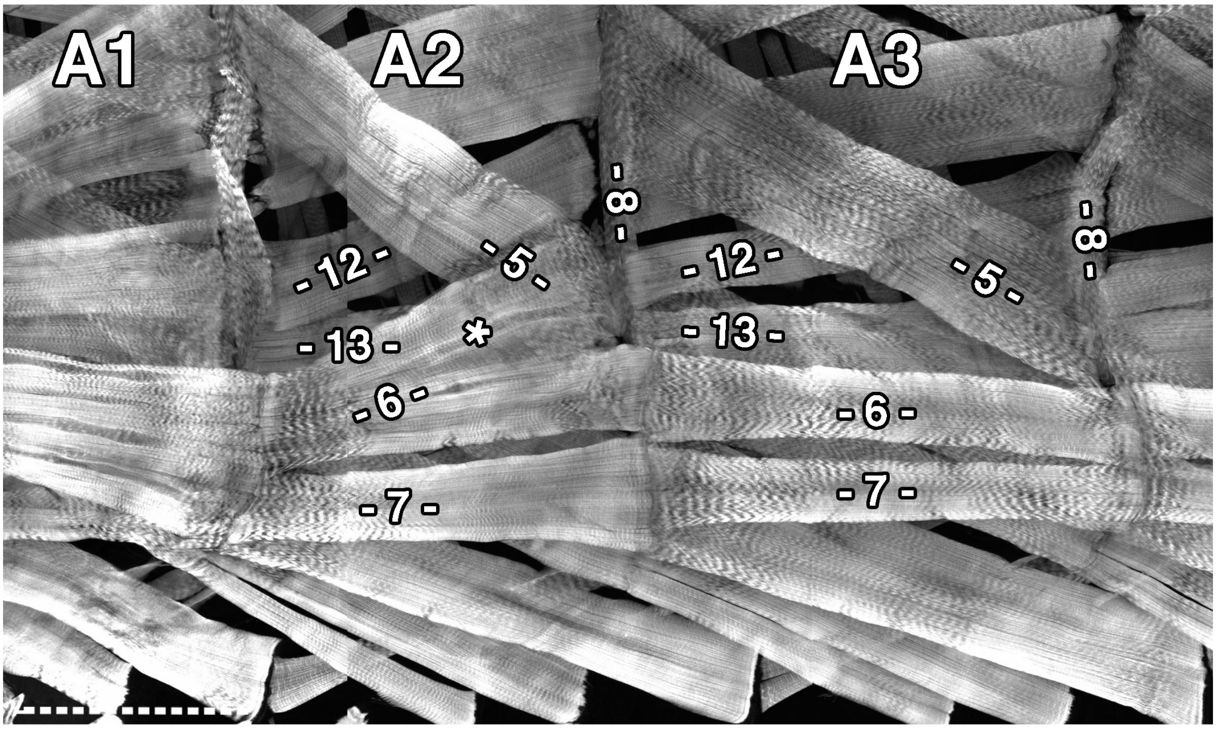

Suppl. Fig. S3

Supplementary Fig. S3

Confocal image (projection) of ventral muscle groups in abdominal hemisegments A1 to A3 of a dissected third instar *Ceratitis capitata* larva stained with phalloidin to label muscle fibres. (Left) Muscle 6 in A2 is tightly attached to a more externally located muscle structure (asterisk) that projects towards M8 but could not be fully separated from M6 in image stacks, giving M6 a triangular shape and much wider appearance.

(Right) Muscle pattern in abdominal hemisegment A3, which lacks a broader M6 fibre.

Ventral muscles M6-M7, M12-M13 and lateral muscle M5 and M8 are numbered. Dorsal is up and anterior left. Dashed line indicates the ventral midline.

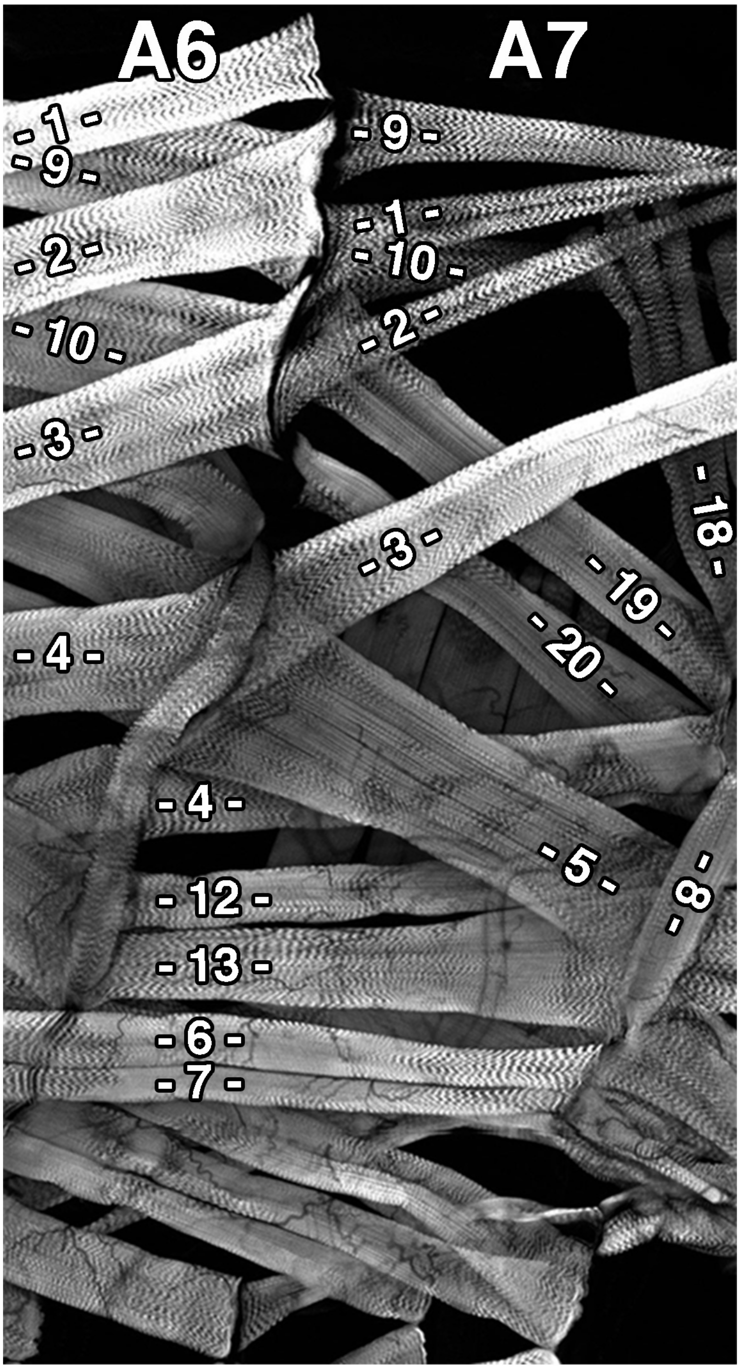

Suppl. Fig. S4

Supplementary Fig. S4

Confocal image (projection) of the muscle pattern in abdominal hemisegments A6 to A7 of a *Ceratitis capitata* third instar larva.

(Left) Stereotyped muscles pattern in of A6. A subset of dorsal muscles are numbered.

(Right) Muscle pattern in abdominal hemisegment A7, showing a rearrangement of dorsal-most muscles M1-M2 and M9-M10. M10 is only partially visible due to incomplete projection. M13 is clearly thicker compared to adjacent muscles M12 and M6.

Dorsal is up and anterior left.
